# Relief for the Medical Profession of Charleston

**Published:** 1886-10

**Authors:** 


### RELIEF FOR THE MEDICAL PROFESSION OF CHARLESTON.

Having received a letter from one of the prominent physicians of Charleston representing the profession of that city as great sufferers by the recent earthquake, we feel prompted to present an appeal to our colleagues of Atlanta and elsewhere in behalf of our afflicted brothers.

While medical men are expected to render all needful assistance to those who are injured, and to attend the sick amongst the population under great disadvantages, their own families undergo all the deprivations of others in an aggravated form, owing to the necessary absence of their natural protectors.

Besides the individual losses and sacrifices to which the medical men of Charleston have been subjected, a great calamity has come upon the profession in the damage done to the medical college building, in whose halls so many practitioners of the South have received valuable instruction from the great lights of the profession in former years.

It is hoped that every physician who has attended lectures in the Medical College of South Carolina, at Charleston, whether recently or in bygone days, will esteem it a privilege to contribute according to his means towards the restoration of the old college building, and that others may cheerfully aid the sufferers among the families of physicians.

We will receive and forward any contributions which may be sent us, and make proper acknowledgments of the same in THE JOURNAL.

